# Optimal reference genes for gene expression analysis in polyploid of *Cyprinus carpio* and *Carassius auratus*

**DOI:** 10.1186/s12863-020-00915-6

**Published:** 2020-09-17

**Authors:** Wenbin Liu, Xiudan Yuan, Shuli Yuan, Liuye Dai, Shenghua Dong, Jinhui Liu, Liangyue Peng, Minmeng Wang, Yi Tang, Yamei Xiao

**Affiliations:** 1 State Key Laboratory of Developmental Biology of Freshwater Fish, College of Life Sciences, Hunan Normal University, Changsha, 410081 Hunan P.R. China; 2 College of Life Sciences, Hunan Normal University, Changsha, 410081 Hunan P.R. China

**Keywords:** Reference gene, Polyploid fish, *RPS5*, *RPS18*

## Abstract

**Background:**

Reference genes are usually stably expressed in various cells and tissues. However, it was reported that the expression of some reference genes may be distinct in different species. In this study, we intend to answer whether the expression of reported traditional reference genes changes or not in the polyploid fish

**Results:**

By retrieving the mRNA sequencing data of three different ploidy fish from the NCBI SRA database, we selected 12 candidate reference genes, and examined their expression levels in the 10 tissues and in the four cell lines of three different ploidy fish by real-time PCR. Then, the expression profiles of these 12 candidate reference genes were systematically evaluated by using the software platforms: BestKeeper, NormFinder and geNorm.

**Conclusion:**

The 28S ribosomal protein S5 gene (*RPS5*) and the ribosomal protein S18 gene (*RPS18*) are the most suitable reference genes for the polyploid of *Cyprinus carpio* and *Carassius auratus*, demonstrated by both of the tissues and the cultured cells.

## Background

Reference genes, generally known as the house-keeping genes, are a type of genes not only stably expressed in various cells but also less affected by external factors [1, 2]. A number of reference genes have been widely used in molecular biology research, such as the beta-actin (*β-actin)*, beta-tubulin *(β-tubulin),* elongation factor 1-alpha (*EF1-α),* Glyceraldehyde-3-phosphate dehydrogenase (*GAPDH)*, ribosomal protein S18 (*RPS18)*, etc. [3–7]. However, it was reported that the expression of some traditional reference genes could change in varying degrees in various species or under different experimental conditions [8–11]. For example, *β-actin* was the most common reference gene, and was stablely expressed in carrot (*Daucus carota*) under the abiotic stress and hormone stimuli, but it is not true in parsley even under the same experimental conditions [12, 13]. Similarly, the expression of *β-tubulin* was not stable during fruit development in cherry (*Cerasus pseudocerasus*) [14], and in different tissues of *Siniperca chuatsie* [15]. Although *GAPDH* was one of the most stable reference genes in the context of whitefly *Bemisia tabaci* (Asia I) thermal stress [10], Zhang et al found that *it* was distinctly expressed in different tissues of Spanish mackerel [16].

Polyploids are the organisms containing three or more complete sets of chromosomes [17]. They are widespread in plants [18–23], and around two hundreds of polyploids have been reported in insect and vertebrate [24–28]. Moreover, the polyploidy can be also found in cells and tissues of diploid organisms, such as muscle tissues, megakaryocytes, and hepatocytes [29, 30]. Polyploidization leads to chromosome doubling and genome structural variation [31–33]. Adams et al. described some significant changes in reference gene expression and the silencing of some homologous genes in different organs of heteropolyploid cotton [34]. We reasoned that polyploidy might affect the stable expression of conventional reference genes. Therefore, to reduce errors and ensure the authenticity and reliability of data, it is necessary to choose the most appropriate reference genes for different ploidy cells or organisms.

The tetraploid hybrid of *C. auratus* (♀) × *C. carpio L.* (♂), and the triploid hybrid of *C. auratus* (♀) × tetraploid hybrid (♂), generated by hybridization of *Carassius auratus (C. auratus*) and *Cyprinus carpio (C. carpio)*, have important theoretical significance in polyploid animal research and remarkable economic benefits [35–37]. To investigate whether there were any changes in the expression of reference genes in varied ploidy fish, we obtained mRNA sequencing (seq) data of the diploid, triploid, and tetraploid fish from the NCBI SRA database [38–41], then chosed 12 candidate reference genes, and examined their expression in 10 tissues and four cell lines of three different ploidy fish by real-time PCR. Finally, we identified the most suitable reference genes for polyploid fish using stability evaluation tools, such as the BestKeeper, NormFinder and geNorm.

## Results

### Validation of reference genes by transcriptome analysis

We chosed 12 commonly used reference genes from our literature search. We retrieved the data of twelve candidate genes shown in Table 1 from the transcriptome. We assigned those genes with an adjusted *P*-value < 0.05 and |log2FoldChange| > 1.5 found by DE Seq as differentially expressed [42, 43]. Fold change refers to the ratio of expression difference between two samples. In the polyploidy tissues, the |log2FoldChange| of *β-actin* between the triploid hybrid (3 N) and the diploid *C. auratus* (2 N) was greater than 1.5, and the *P*-value of *B2M* was less than 0.05; while between the tetraploid hybrid (JL4N) and the diploid *C. auratus*, the |log2FoldChange| of *RPL7, RPLP2, RPL13α, GAPDH* were all greater than 1.5, and the *P*-values of *RPS5, RPL7, RPL13α, EF1-α, GAPDH* were less than 0.05. In cultured cells*,* the |log2FoldChange| of candidate genes between the tetraploid hybrid cultured fin cells and diploid *C. auratus* cultured fin cells were all less than 1.5, the *P* values of *RPS18, RPLP2, RPL13α, B2M* were less than 0.05; The |log2FoldChange| of candidate genes between SP4N cells (SP4N) and diploid *C. auratus* cultured fin cells were all less than 1.5, the *P*-value of *B2M* was less than 0.05. Based on the transcriptome data, we found that the expression of some traditional reference genes was unstable in polyploidy, such as the *β-actin*.

**Table 1:** Transcriptome analysis of candidate reference genes

Gene	Tissues							Cultured cells						
	Tissues (FPKM)			log2FoldChange		*P* value		Cells (FPKM)			log2FoldChange		*P* value	
	2 N	3 N	JL4N	3 N/2 N	JL4N/2 N	3 N/2 N	JL4N/2 N	2 N	JL4N	SP4N	JL4N/2 N	SP4N/2 N	JL4N/2 N	SP4N/2 N
*RPS5*	849.24	678.32	473.93	−0.32	− 0.84	0.10	0.01	849.24	856.28	667.33	0.01	−0.35	0.93	0.05
*RPS18*	2068.00	1038.73	1183.93	−0.99	− 0.80	0.32	0.31	1219.08	1758.57	974.64	0.53	−0.32	0.04	0.22
*RPL7*	911.01	862.83	224.46	−0.08	−2.02	0.68	0.00	911.01	1107.82	754.63	0.28	−0.27	0.14	0.10
*RPLP2*	238.01	372.57	949.56	0.65	2.00	0.23	0.14	238.01	442.26	153.76	0.89	−0.63	0.00	0.10
*RPL13α*	982.73	715.70	251.13	−0.46	−1.97	0.15	0.00	1083.97	1434.20	689.13	0.40	−0.65	0.01	0.02
*β-actin*	578.00	128.05	435.73	−2.17	−0.41	0.14	0.57	1212.30	2115.03	1517.57	0.80	0.32	0.08	0.33
*EF1-α*	2074.67	2286.30	855.60	0.14	−1.28	0.65	0.02	3693.40	4006.77	1957.93	0.12	−0.92	0.79	0.17
*DDX5*	68.30	88.85	41.80	0.38	−0.71	0.23	0.26	74.10	90.77	80.90	0.29	0.13	0.08	0.35
*β-tubulin*	29.80	51.30	33.67	0.78	0.18	0.09	0.71	240.73	273.17	294.77	0.18	0.29	0.64	0.45
*hprt1*	26.93	47.40	20.93	0.82	−0.36	0.27	0.64	28.27	15.53	19.10	−0.86	−0.57	0.33	0.47
*B2M*	96.53	263.95	89.73	1.45	−0.11	0.02	0.91	200.10	100.97	95.13	−0.99	−1.07	0.02	0.02
*GAPDH*	2324.47	1332.75	539.83	−0.80	−2.11	0.32	0.04	0.70	0.93	0.67	0.41	−0.06	0.63	0.93

### Analysis of qPCR cycle threshold value

**Fig. 1:**
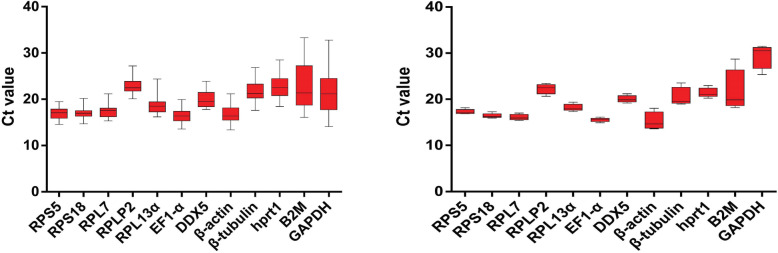
The qPCR cycle threshold (Ct) values of candidate reference genes in tissues (Left) and cultured cells (Right) of different ploidy fish. The red area represented the range of Ct value variation

First, we confirmed the ploidy level and DNA content of each sample by flow cytometry (Figure S1, S2). Then we examined the expression of twelve candidate reference genes in tissues and cultured cells of different ploidy fish using Real-time quantitative PCR (qPCR). The qPCR cycle threshold (Ct) values were used to represent the level of mRNA transcription [44]. Based on the data of qPCR (Figure S3, S4), the Ct values for some of the selected reference genes varied significantly (Fig. 1). The *RPS5*, *RPS18*, *RPL7*and *EF1-α* were highly expressed in polyploidy tissues, while *RPS18*, *RPL7*,*EF1-α* and *β-actin* exhibited high expression in cultured cells. These results showed that in polyploid tissues, the expression of *RPS5*, *RPS18*, and *RPL7* was more stable than that of *B2M* and *GAPDH*. On the other hand, in cultured cells of different polyploid, the expression of *EF1-α*, *RPS5* and *RPS18* was stable, while that of *B2M* and *GAPDH* was not.

### Bestkeeper analysis

The Bestkeeper program was further used to validate the relatively stable reference genes [5]. BestKeeper ranks the reference genes according to the stability of gene expression based on the two parameters: the standard deviations (SD) and the coefficient of variance (CV) of expression levels. The lower SD and CV values, the higher stability [45]. Among the 10 different tissues of tetraploid hybrid and triploid hybrid *C. auratus*, only the SD value of *RPS18* gene was less than 1, while the SD values of the other 11 genes were all greater than 1, indicating they were not suitable for the use of reference gene. The order of expression stability from high to low was *RPS18, RPS5, RPL7, RPL13α, EF1-α, RPLP2, DDX5, β-actin, β-tubulin, hprt1, GAPDH,* and *B2M* in the 10 tissues of different polyploid fish (Table 2). In the cultured cells, the SD values of *β-actin*, *β-tubulin*, *GAPDH*, *B2M* were all higher than 1, suggesting they were not ideal candidates of reference genes. The expression stability ranking from high to low was *EF1-α, RPS5, RPS18, RPL7, DDX5, RPL13α, RPLP2, hprt1, β-actin, β-tubulin, GAPDH and B2M* (Table 2)*.*

**Table 2:** The expression stability of candidate reference genes in tissues and cultured cells of different ploidy fish assessed by the Bestkeeper software

Gene	Tissues			Cultured cells		
	SD	CV	Rank	SD	CV	Rank
*RPS5*	1.06	6.24	2	0.38	2.19	2
*RPS18*	0.18	4.75	1	0.4	2.45	3
*RPL7*	1.15	6.58	3	0.44	2.73	4
*RPLP2*	1.3	5.7	6	0.78	3.5	7
*RPL13α*	1.23	6.61	4	0.57	3.15	6
*EF1-α*	1.3	7.85	5	0.3	1.9	1
*DDX5*	1.4	7.04	7	0.56	2.79	5
*β-actin*	1.54	9.24	8	1.46	9.58	9
*β-tubulin*	1.76	8.11	9	1.54	7.54	10
*hprt1*	1.87	8.22	10	0.8	3.77	8
*B2M*	4.83	20.71	12	3.49	16.19	12
*GAPDH*	3.91	18.34	11	2.05	6.95	11

### NormFinder analysis

**Fig. 2:**
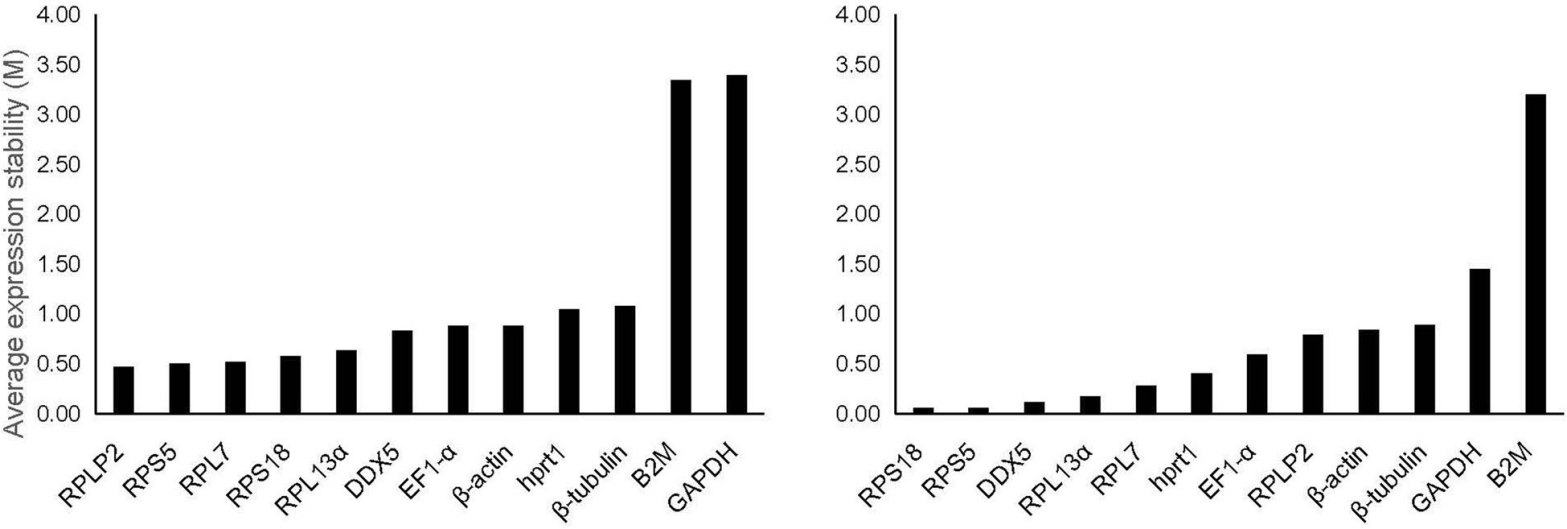
The expression stability of candidate reference genes in the tissues (Left) and cultured cells (Right) of different ploidy fish assessed by the NormFinder software

The NormFinder software applies a mathematical model to estimate the variation of the candidate reference genes (intra- and inter-group expression variations) [46]. To determine stable reference genes, we analyzed the data obtained from the qPCR using NormFinder [47]. As shown in Fig. 2, in the 10 tissues of different polyploid fish, the ranking of expression stability from high to low was *RPLP2, RPS5, RPL7, RPS18, RPL13α, DDX5, EF1-α, β-actin, hprt1, β-tubulin, B2M,* and *GAPDH*. In the cultured cells of different polyploid, the ranking of stability was, from high to low, *RPS18, RPS5, DDX5, RPL13α, RPL7, hprt1, EF1-α, RPLP2, β-actin, β-tubulin, GAPDH,* and *B2M* (Fig. 2).

### GeNorm analysis

**Fig. 3:**
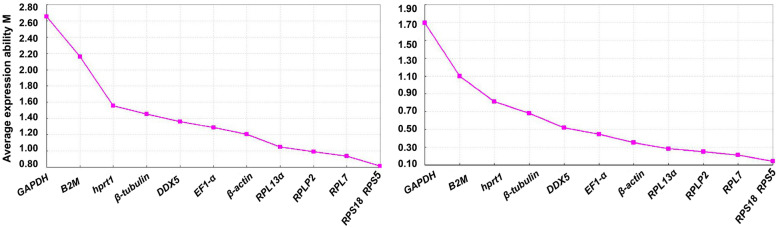
The stability of candidate reference genes in tissues (Left) and cultured cells (Right) of different ploidy fish determined by GeNorm analysis

GeNorm calculates value M based on the pairwise variation between an individual gene and all other tested candidate genes. The M value is inversely proportional to the stability of particular reference genes. In general, if the M value is less than 1.5, it can be used as an alternative reference gene [12]. The program can also determine the optimal number of reference genes by pairwise difference analysis of normalized factors [45, 48]. As shown in Fig. 3, in the 10 different tissues of diploid *C. auratus*, triploid hybrid and tetraploid hybrid, the expression stability in descending order was listed by *RPS5, RPS18, RPL7, RPLP2, RPL13α, β-actin, EF1-α, DDX5, β-tubulin, hprt1 B2M* and *GAPDH*. Their M values were given by 0.183, 0.183, 0.936, 0.988, 1.048, 1.208, 1.290, 1.361, 1.455, 1.558, 2.159 and 2.655, respectively In the cultured cells of *C. auratus*, triploid hybrid, tetraploid hybrid and SP4N cell line, the M values of *RPS5, RPS18, RPL7, RPLP2, RPL13α, β-actin, EF1-α, DDX5, β-tubulin, hprt1, B2M,* and *GAPDH* were 0.143, 0.143, 0.211, 0.520, 0.250, 0.684, 0.447, 0.281, 0.812, 0.350, 1.696, and 1.100, respectively (Fig. 3).

**Fig. 4:**
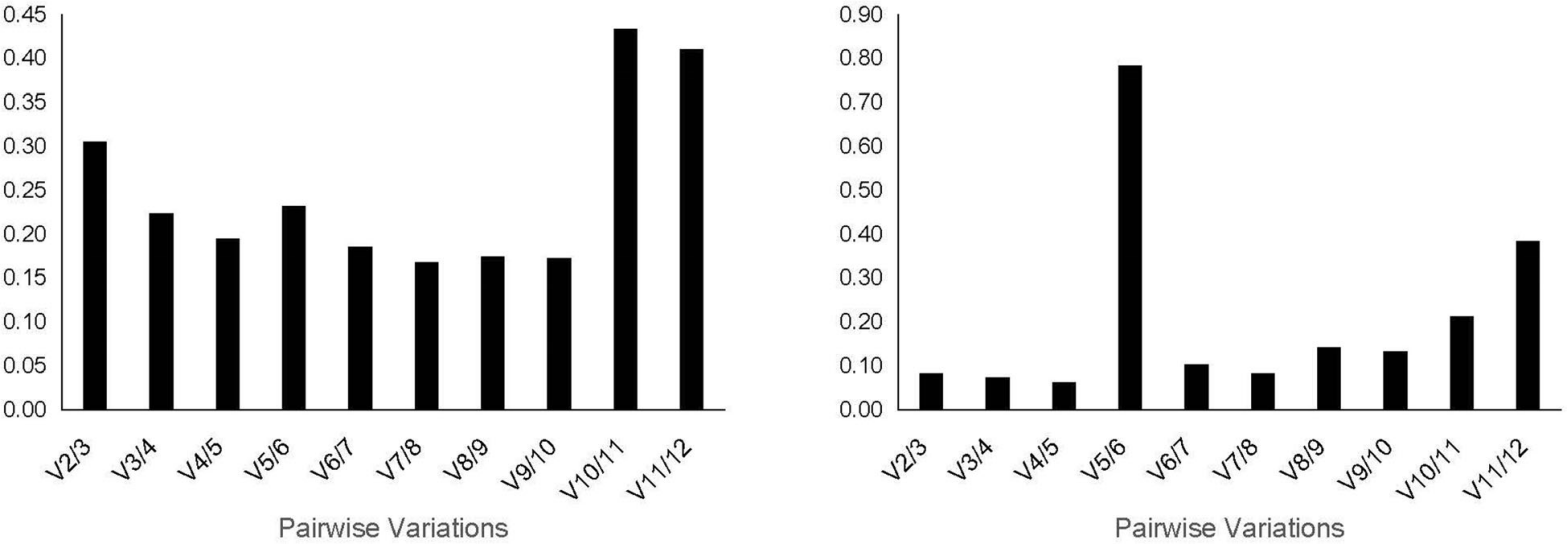
Optimal number of reference genes for normalization of gene expression in tissues (Left) and cultured cells (Right). The geNorm was used to calculate the pairwise variation (Vn/Vn + 1, the “n” represents the number of reference genes)

In addition, the pairwise variation V calculated by geNorm was also used to determine the minimum number of the reference genes for optimal normalization: If the Vn/Vn + 1 value was < 0.15, the number of optimal reference genes was n, and if the Vn/Vn + 1 value was > 0.15, the optimal number of reference genes was n + 1 [10, 11]. As shown in Fig. 4, the Vn/(n + 1) values in the polyploid tissues were all greater than 0.15, while those of polyploid cultured cells, the V2/3 were less than 0.15. The results suggested that two reference genes should be combined for optimal normalization polyploid cultured cells, while it was necessary to adjust the conditions accordingly to determine the appropriate number of reference genes.

## Discussion

In this study, 12 traditional reference genes were selected as candidate reference genes after literature search [3–7], then subjected to transcriptome analysis. These 12 relatively stable candidate reference genes in polyploid of *Cyprinus carpio* and *Carassius auratus* were evaluated using BestKeeper, NormFinder and geNorm, three reference gene stability analysis softwares that are widely used in the reference gene selection in species such as animals, micro-organisms and plants [45, 47–50]. For the polyploid tissues, the top three stable reference genes evaluated by BestKeeper were *RPS18, RPS5* and *RPL7*, those by NormFinder were *RPLP2, RPS5* and *RPL7*, and those by geNorm were *RPS5, RPS18* and *RPL7*. As for the polyploid cultured cells in vitro, the top three stable reference genes evaluated by BestKeeper were *EF1-α, RPS5* and *RPS18*, those by NormFinder were *RPS18, RPS5* and *RPLP2*, and those by geNorm were *RPS5, RPS18* and *RPL7*. Our data showed that the expression levels of *RPS5* and *RPS18* were relatively stable not only in the polyploid fish tissues in vivo but also in the cultured cells in vitro.

For some reasons, such as extreme conservativation of evolution, highly and stably expression in different types of cells and tissues, several components of ribosomes were selected as reference genes [47, 51–55]. In eukaryotic cells, the ribosomal RNA (rRNA) genes exist in multiple copies, however, without poly (A) tail, rRNA could not be reversely transcribed in cDNA synthesis using oligo (dT) as a primer [53, 54, 56]. Both *RPS5* and *RPS18* are the ribosomal protein genes, which were highly expressed in the eukaryotic organisms [52]. The RPS5 was closely related to transcription elongation factor-3 and DNA connection protein II, and its expression was highly conserved [47, 57–63]. RPS18, a highly conserved member of the nuclear protein S13 superfamily, is one of the constituent proteins of the eukaryotic ribosomal 40S subunit, and stably expressed in different tissues [34, 64–68]. Therefore, with the high expression level and best stability, we selected *RPS5* and *RPS18* as the suitable reference genes in the polyploid of *Cyprinus carpio* and *Carassius auratus*.

Moreover, in line with the settings of the geNorm software, in polyploid cultured cells, the pairwise variation analysis showed that the V2/3 < 0.15, but all Vn/Vn + 1 values were greater than 0.15 in the polyploid tissues. Cao et al encountered a similar problem in the selection of reference genes of Ruditapes philippinarum [69, 70]. It indicated that the two reference genes were sufficient for the gene expression normalization of the polyploid cultured cells, but it was necessary to adjust the conditions accordingly to determine an appropriate number of the reference genes in the polyploid tissues.

## Conclusion

In this study, we systematically evaluated the expression profiles of 12 selected candidate reference genes of the polyploid fish using BestKeeper, NormFinder and geNorm, and determined a comprehensive ranking of these genes. We confirmed that *RPS5* and *RPS18* were the most stable reference genes across different tissues and cultured cells for polyploid of *Cyprinus carpio* and *Carassius auratus*. These reference genes identified in this study will become useful tools for the molecular biology of polyploid fish.

## Methods

### Ethics statement

All sampling procedures were conducted in accordance with the standards and ethical guidelines established by the Animal Ethical Review Committee, Hunan Normal University, Changsha, China.

### Sample preparation

Fish, including *C.auratus* (2 N), tetraploid hybrid (JL4N) of *C.auratus* (♀) × *C. carpio L*.(♂), and triploid hybrid (3 N) of *C. auratus* (♀) × tetraploid hybrid (♂), were bred and fed in pools under the same water temperature, dissolved oxygen content, and for aging conditions at the Engineering Research Center of Polyploid Fish Breeding and Reproduction of the State EducationMinistry, China. Three individuals (1-year-old) of each species were collected for further study. Under aseptic conditions, ten tissues, e.g. liver, kidney, heart, spleen, brain, caudal fin, skin, muscle, intestine and gonad, were dissected from above three different ploidy fish, respectively.

Cultured cells were obtained from the caudal fin of *C. auratus*, triploid hybrid, and tetraploid hybrid. The SP4N cell line (SP4N) was obtained from *C. auratus* caudal fin cells induced by the c-Jun N-terminal kinase inhibitor SP600125 in vitro [39]. The cells were cultured in the Dulbecco’s modified Eagles medium (DMEM; Sigma) supplemented with 100 U/ml penicillin, 100 μg/ml streptomycin (Invitrogen, Carlsbad, CA, USA), 10% fetal bovine serum (FBS, Invitrogen, Carlsbad, CA, USA), 0.1% 2-mercaptoethanol (2-ME, Invitrogen, Carlsbad, CA, USA), 1 mM sodium pyruvate (Invitrogen, Carlsbad, CA, USA), and 1 mM nonessential amino acids (Invitrogen, Carlsbad, CA, USA). Cells were grown in 5%(v/v) CO_2_ at 28 °C.

### RNA isolation

Total RNA was isolated from tissues and culture cells of three different ploidy fish, using Trizol Reagent (Invitrogen, USA) following manufacturer protocols [71]. The quality of total RNA was detected by 1% agarose gel electrophoresis and nucleic acid analyzer [5].

### Transcriptome data obtaining and analysis

We obtained mRNA sequencing (RNAseq) data of the liver tissue of diploid *C. auratus*, triploid hybrid and tetraploid hybrid from the NCBI Sequence Read Archive (SRA) database (Accession numbers: diploid *C. auratus*: SRR538839, SRR542431; triploid hybrid: SRR9185090, SRR9203584; tetraploid hybrid: SRR1535135, SRR1536195;) [38, 39]. And the RNAseq data of the cultured cells of diploid *C. auratus*, tetraploid hybrid, and SP4N cells also were obtained from the NCBI SRA database (Accession number: cultured cells of diploid *C. auratus:* SRR7640868, SRR7640869; cultured cells of tetraploid hybrid: SRR7640866, SRR7640867; SP4N cells: SRR9964682, SRR9964683) [40, 41].

All accessions and biological replicates were normalized using the method of DESeq2 [71], and three biological replicates were used in each analysis. The negative effects of background noise were eliminated by removing those low read counts of transcripts (≤2) from the datasets. The values of fragments per kilobase of transcript per million mapped reads (FPKM) were used to analyze differential expression (DE) between diploid and triploid or diploid and tetraploid [72, 73].

The FPKM threshold 0.1 was set to determine whether the gene was expressed, where transcripts with FPKM ≤0.1 were defined as no expression [74]. The higher the FPKM value, the stronger the gene expression. For polyploidy tissues, we used the transcriptome of diploid *C. auratus* liver as a reference, calculated the log2FoldChange between triploid hybrid and diploid, and log2FoldChange between tetraploid hybrid and diploid. For polyploid cultured cells, the transcriptome of diploid *C. auratus* cultured fin cell was used as a reference, we calculated the log2FoldChange between tetraploid hybrid cultured fin cells and diploid cultured fin cells, and log2FoldChange between SP4N cells and diploid cultured fin cells. The difference in gene expression was evaluated according to log2Fold Change [62], which was calculated based on the FPKM value. As to the transcriptome data constructed in our laboratory, we cacluated the FPKM values of some common reference genes and performed multiple sequence alignment on these genes. Alignments of multiple sequences were used to ensure that the sequence similarity of the ortholog genes in different ploidy fish was above 99%.

### Real-time quantitative PCR (qPCR)

The reference genes were identified by bioinformatics analysis. The qPCR primers were designed using the Olig7 software and listed in Table S2. The cDNAs were synthesized according to the manual of the TaKaRa reverse transcription kit. Genomic DNA was first eliminated from the RNA. For reverse transcription, 1 μg RNA was used for each reaction. For PCR, the five-fold dilution series of template cDNA was used. Each biological replicate was run in triplicate on a Bio-Rad CFX-96 system. Each 10 μL qPCR reaction mixture contains 1μLof cDNA (0.01 μg RNA), 5 μL of Brilliant SYBR Green QPCR Master Mix and primer pair. SetNTC (No template control) negative controls for each sample, negative control reactions were included in all assays by substituting water for template DNA to confirm that no DNA contamination was present in RNA samples [10, 51, 52]. Each thermal cycle consisted of an initial polymerase activation step at for 50 °C for 2 min and 95 °C for 10 min, followed by 40 cycles at 95 °C for 15 s, at 60 °C for 1 min. Afterwards, melting curves were generated to confirm a single gene-specific peak and to detect primer dimer formation by heating the samples stepwise from 60 °C to 95 °C while continuously monitoring the fluorescence. For each sample, the qPCR analysis was performed on three biological replicates. The specificity of all qPCR was confirmed by melting curve analysis of amplification products (Figure S5) [75]. To evaluate the expression levels of all candidate references across different tissues and cells of different ploidy fish, comparative analysis of all acquired Ct values for each gene, lower Ct values represents higher mRNA transcript levels [14].

### Evaluation of gene expression stability

The Ct values calculated from qPCR data were used for further analyses. The stability of reference genes was evaluated by the Bestkeeper, NormFinder and geNorm [47, 49, 76]. The BestKeeper program is an excel spreadsheet with built-in formulas, the input C*P* value is the average Ct value obtained from each biological replicate in qPCR. For NormFinder and geNorm, the quantification cycle (Ct) values were transformed into relative quantities using the formula 2^-△Ct^, whereΔCt = each corresponding Ct value-the minimum Ct value. NormFinder applies a mathematical model to estimate the variation of the candidate reference genes. GeNorm calculated the expression stability measurement (M-value), which was based on the average variation in the expression level of a particular gene against that of all the control genes [14]. This programme also evaluates the pairwise variation (Vn/Vn + 1) to determine the optimal number of genes required for accurate normalization of qPCR data. Finally, a comprehensive analysis and comparison of the results of three software to evaluate the stability of candidate reference genes in different tissues and cells of different ploidy fish.

## Supplementary information

**Additional file 1: Figure S1.** Fish DNA content detection.

**Additional file 2: Figure S2.** Cell DNA content detection.

**Additional file 3: Figure S3.** Real-time quantitative CT values of candidate reference genes in ten different tissuesof different ploidy fish.

**Additional file 4: Figure S4.** Real-time quantitative CT values of candidate reference genes in cultured cells of different ploidy fish.

**Additional file 5: Table S1.** Evaluation of candidate reference genes in tissues and cultured cells of different ploidy fish.

**Additional file 6: Table S2.** qPCR primers for candidate reference genes.

**Additional file 7: Figure S5.** Primer melting curves.

## Data Availability

The raw data supporting our findings can be found in the NCBI SRA database under the accession number (Accession number: the liver tissue of diploid *C. auratus*: SRR538839, SRR542431; the liver tissue of triploid hybrid: SRR9185090, SRR9203584; the liver tissue of tetraploid hybrid: SRR1535135, SRR1536195; cultured fin cells of diploid *C. auratus*: SRR7640868, SRR7640869; cultured fin cells of tetraploid hybrid: SRR7640866, SRR7640867; SP4N cells: SRR9964682, SRR9964683).
