# Supplementary figures and images for: Optimal reference genes for gene expression analysis in polyploid of Cyprinus carpio and Carassius auratus
Source: BMC Genet. 2020 Sep 17;21:107. doi: 10.1186/s12863-020-00915-6 (PMC7499967; doi:10.1186/s12863-020-00915-6)

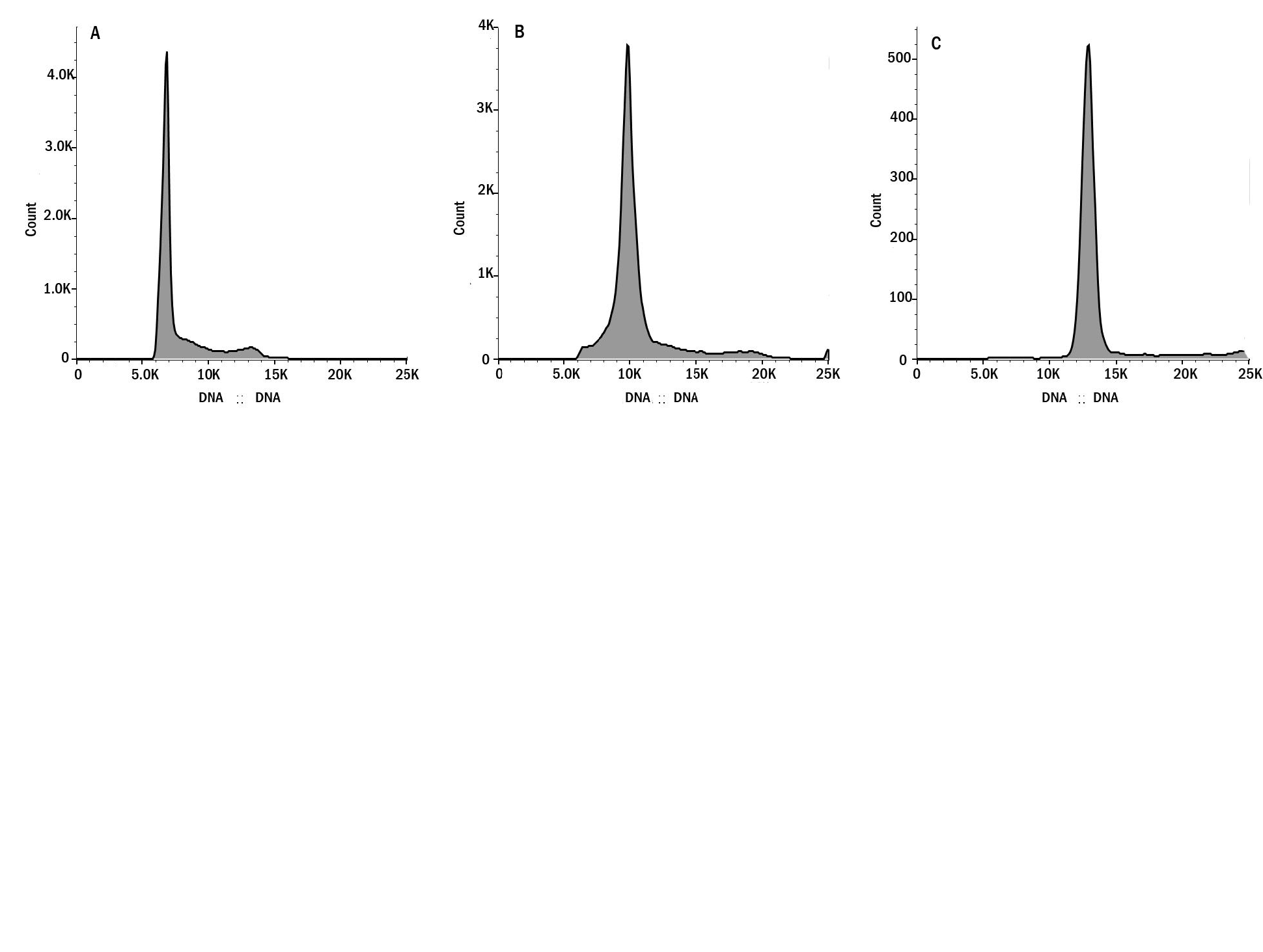


**Figure S1** Fish DNA content detection. (**A**) diploid *C. auratus*, (**B**) triploid hybrid, (**C**) tetraploid hybrid

Supplement: Supplementary file 1 — Additional file 1: Figure S1. Fish DNA content detection. [file 12863_2020_915_MOESM1_ESM.docx]
