# Supplementary material for: Optimal reference genes for gene expression analysis in polyploid of Cyprinus carpio and Carassius auratus
Source: BMC Genet. 2020 Sep 17;21:107. doi: 10.1186/s12863-020-00915-6 (PMC7499967; doi:10.1186/s12863-020-00915-6)
Supplement: Supplementary file 2 — Additional file 2: Figure S2. Cell DNA content detection. [file 12863_2020_915_MOESM2_ESM.docx]

**
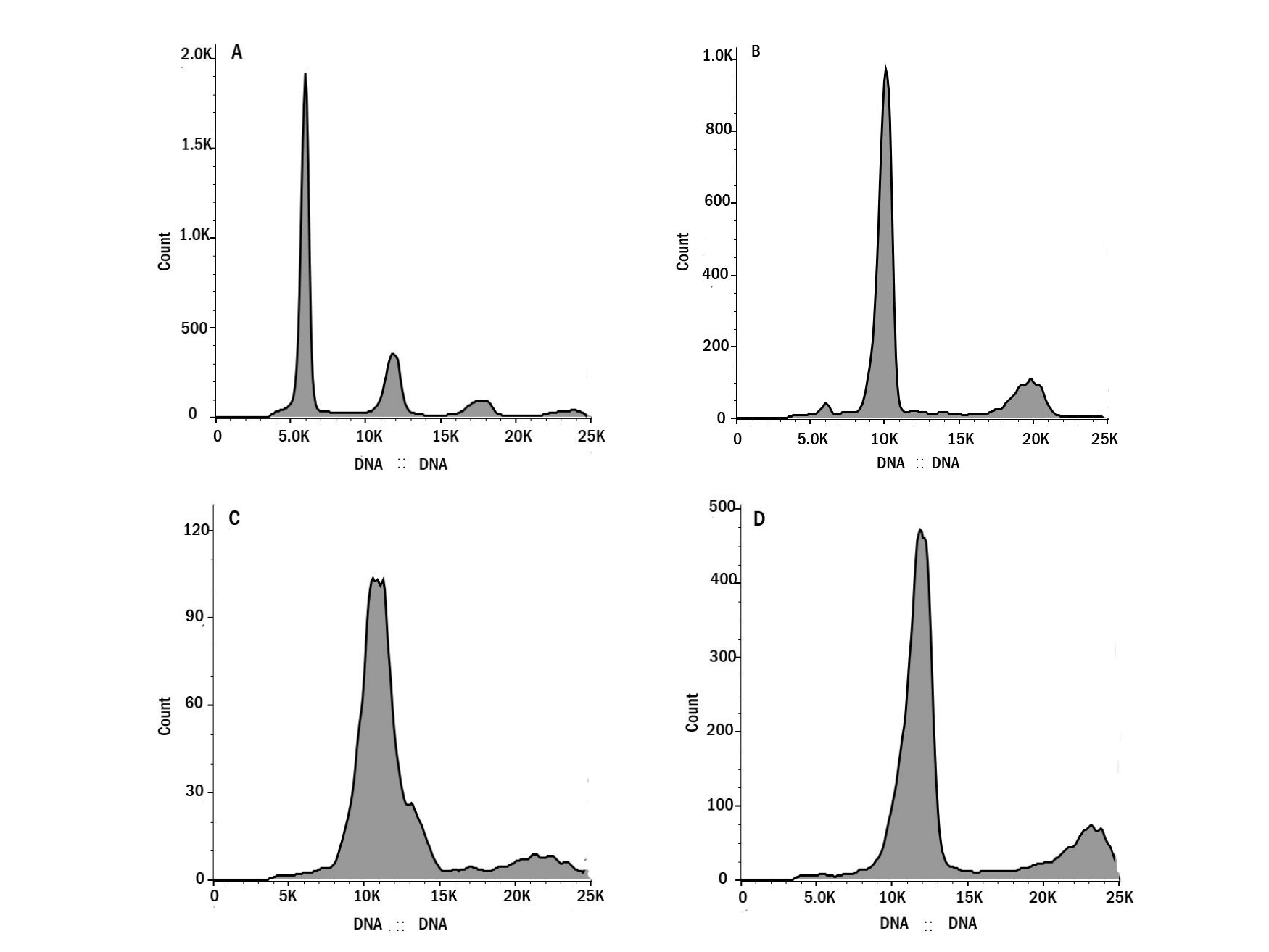
**

**Figure S2** Cell DNA content detection. (**A**) cultured fin cells of diploid *C. auratus*, (**B**) cultured fin cells of triploid hybrid, (**C**) cultured fin cells of tetraploid hybrid, (**D**) SP600125-induced tetraploid (SP4N) cells
