# Supplementary material for: Optimal reference genes for gene expression analysis in polyploid of Cyprinus carpio and Carassius auratus
Source: BMC Genet. 2020 Sep 17;21:107. doi: 10.1186/s12863-020-00915-6 (PMC7499967; doi:10.1186/s12863-020-00915-6)
Supplement: Supplementary file 4 — Additional file 4: Figure S4. Real-time quantitative CT values of candidate reference genes in cultured cells of different ploidy fish. [file 12863_2020_915_MOESM4_ESM.docx]

**
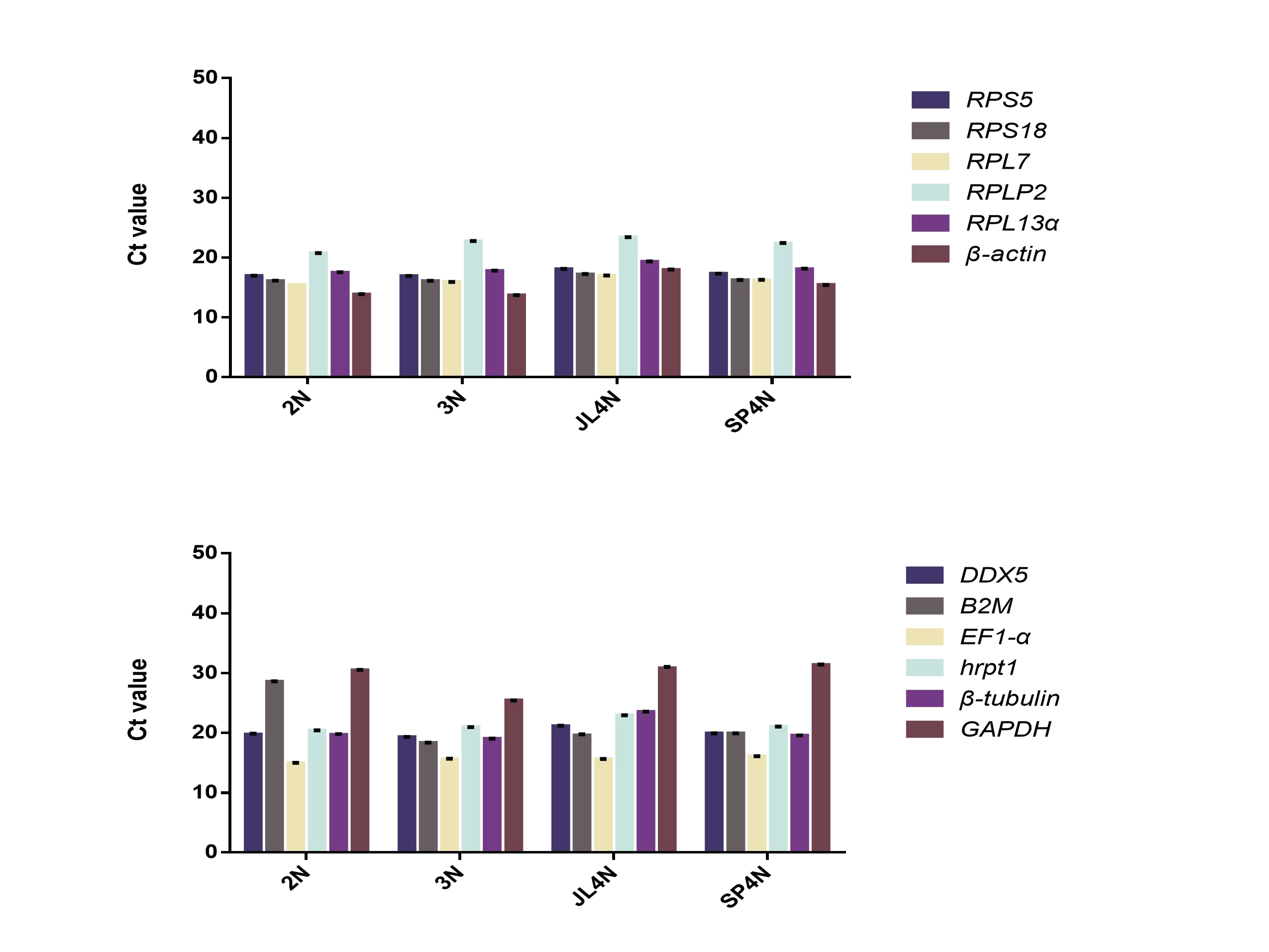
**

**Figure S4** Real-time quantitative CT values of candidate reference genes in cultured cells of different ploidy fish
