# Supplementary material for: Optimal reference genes for gene expression analysis in polyploid of Cyprinus carpio and Carassius auratus
Source: BMC Genet. 2020 Sep 17;21:107. doi: 10.1186/s12863-020-00915-6 (PMC7499967; doi:10.1186/s12863-020-00915-6)
Supplement: Supplementary file 5 — Additional file 5: Table S1. Evaluation of candidate reference genes in tissues and cultured cells of different ploidy fish. [file 12863_2020_915_MOESM5_ESM.docx]

**Table S1** Evaluation of candidate reference genes in tissues and cultured cells of different ploidy fish

| Gene | Tissues | | |  | Cultured cells | | |  |
| --- | --- | --- | --- | --- | --- | --- | --- | --- |
|  | Bestkeeper  rank | NormFinder  rank | geNorm  rank | Rank | Bestkeeper  rank | NormFinder  rank | geNorm  rank | Rank |
| *RPS5* | 2 | 2 | 1 | 1(5) | 2 | 1 | 1 | 1(4) |
| *RPS18* | 1 | 4 | 1 | 2(6) | 3 | 1 | 1 | 2(5) |
| *RPL7* | 3 | 3 | 2 | 3(8) | 4 | 4 | 2 | 3(10) |
| *RPLP2* | 6 | 1 | 3 | 4(10) | 5 | 2 | 4 | 4(11) |
| *RPL13α* | 4 | 5 | 4 | 5(13) | 6 | 3 | 3 | 5(12) |
| *EF1-α* | 5 | 7 | 6 | 6(18) | 1 | 6 | 6 | 6(13) |
| *DDX5* | 7 | 6 | 7 | 7(20) | 8 | 5 | 5 | 7(18) |
| *β-actin* | 8 | 8 | 5 | 8(21) | 7 | 7 | 7 | 8(21) |
| *β-tubulin* | 9 | 10 | 8 | 9(27) | 9 | 8 | 8 | 9(25) |
| *hprt1* | 10 | 9 | 9 | 10(28) | 10 | 9 | 9 | 10(28) |
| *B2M* | 12 | 11 | 10 | 11(33) | 11 | 10 | 10 | 11(33) |
| *GAPDH* | 11 | 12 | 11 | 12(34) | 12 | 11 | 11 | 12(34) |
