# Supplementary material for: Optimal reference genes for gene expression analysis in polyploid of Cyprinus carpio and Carassius auratus
Source: BMC Genet. 2020 Sep 17;21:107. doi: 10.1186/s12863-020-00915-6 (PMC7499967; doi:10.1186/s12863-020-00915-6)
Supplement: Supplementary file 6 — Additional file 6: Table S2. qPCR primers for candidate reference genes. [file 12863_2020_915_MOESM6_ESM.docx]

**Table S2** Candidate reference gene qPCR primers

| Gene name | Forward Primer | Reverse Primer | Tm | Primer product |
| --- | --- | --- | --- | --- |
| *β-actin* | ATACTCCTGCTTGCTAATCCAC | ATGTACCCTGGCATTGCT | 57 | 174 |
| *GAPDH* | ACATCCCCGTTGAAGTCAC | CTCAATGGCAAGCTTACTGG | 57 | 200 |
| *RPS5* | CACGCCTTTGAGATCATCCAC | TGTCTCCTCACGGTTCCAG | 57 | 128 |
| *RPS18* | TCATGCAGAATCCTCGCCAGT | CACGCAGACCCCAGAAGTGAC | 60 | 183 |
| *EF-1α* | CCCAAGGCTCTCAAATCTGG | GGGAAGAACGTACCACAACC | 58 | 265 |
| *β-tubulin* | TCAAACTCACAACGCCCAC | TGTTCACCGCCAGTTTACGAA | 57 | 126 |
| *RPL13α* | CAAATCTGCGTGTTGGCTTC | ACCGTATCACTTTAGAGCTCC | 57 | 200 |
| *RPLP2* | GCTCTTTCCCTCTACTCGTCCT | GGCCTCAATTCCAACACTCCC | 60 | 180 |
| *RPL7* | ATGGTGTCAGCCCTAAAGTCC | ATCTTGCCAAATCCACGCTTG | 60 | 190 |
| *B2M* | GCTGAAGGATGGCGAGATTC | TTATCCGCCTTTCCTCCACC | 58 | 214 |
| *DDX5* | CAGGATAACCGCAACAGTGAC | ACTGTTGGTTCTGATAGCCTT | 58 | 190 |
| *hprt1* | CTGGACCTCTTCTGTATCCC | CACAGAGCCACGATATGATGC | 57 | 143 |
