# Supplementary material for: Optimal reference genes for gene expression analysis in polyploid of Cyprinus carpio and Carassius auratus
Source: BMC Genet. 2020 Sep 17;21:107. doi: 10.1186/s12863-020-00915-6 (PMC7499967; doi:10.1186/s12863-020-00915-6)
Supplement: Supplementary file 7 — Additional file 7: Figure S5. Primer melting curves. [file 12863_2020_915_MOESM7_ESM.docx]

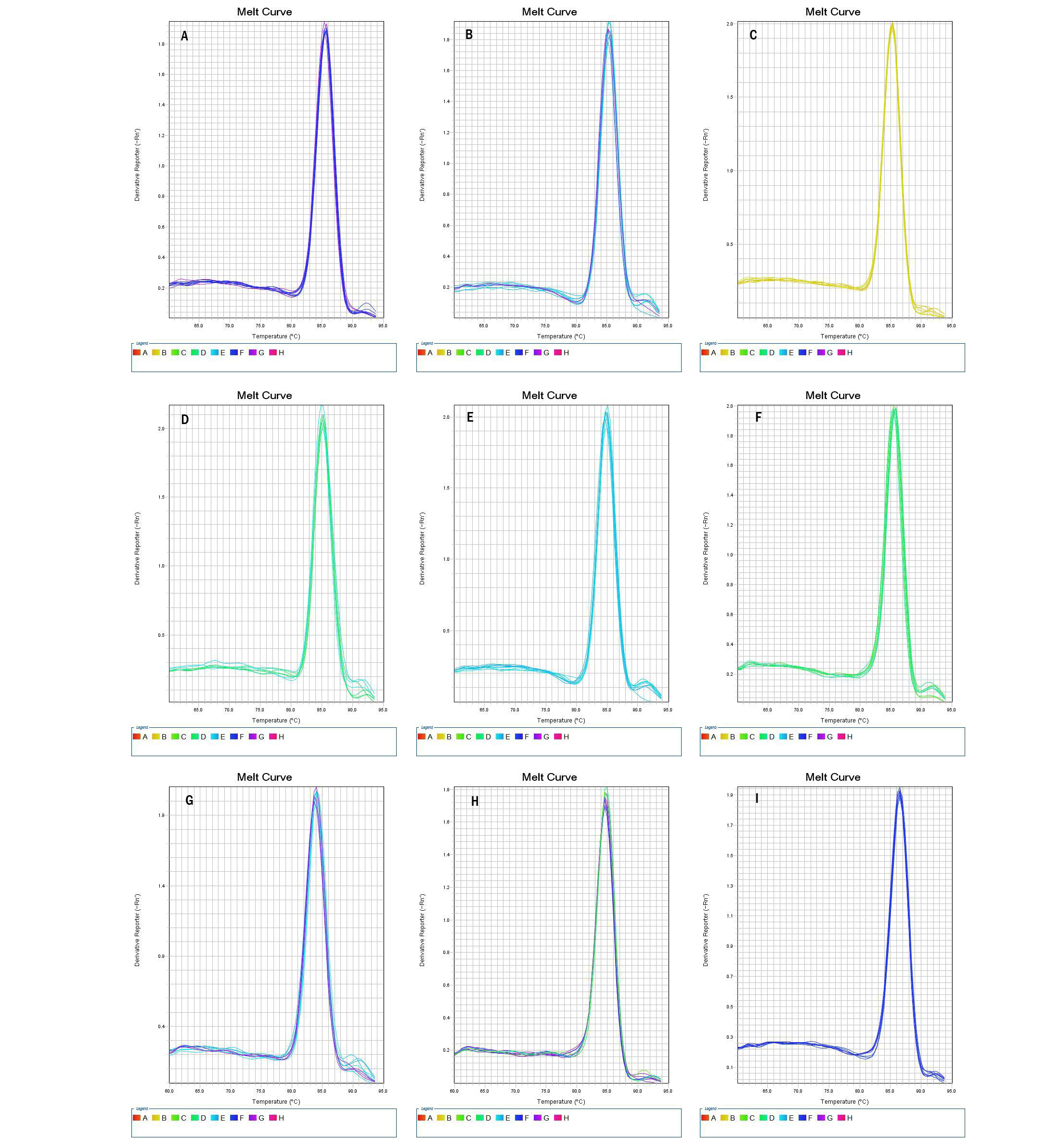


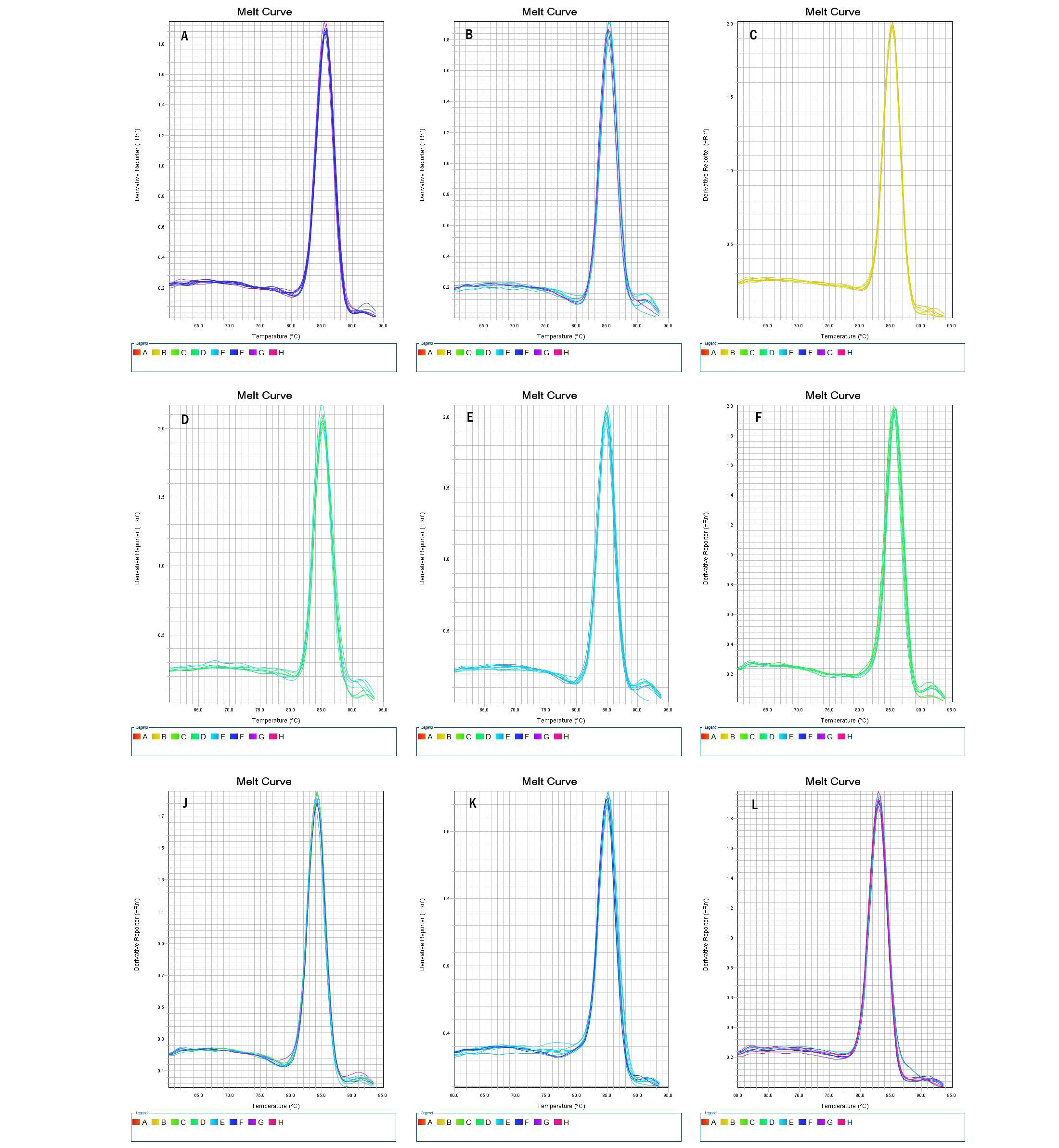


**Figure S5** Primer melting curves. (**A**) *RPS5*, (**B**)*RPS18*, (**C**) *RPL7*, (**D**) *RPLP2*, (**E**) *RPL13α*, (**F**) *EF1-α*, (**G**) *DDX5*, (**H**) *β-actin,* (**I**) *β-tubulin*, (**J**) *hprt1*, (**K**) *B2M*, (**L**) *GAPDH*
